# A guide to *Stenotrophomonas maltophilia* virulence capabilities, as we currently understand them

**DOI:** 10.3389/fcimb.2023.1322853

**Published:** 2024-01-11

**Authors:** Radhika Bhaumik, Nabiha Zumana Aungkur, Gregory G. Anderson

**Affiliations:** Department of Biology, Purdue School of Science, Indiana University Purdue University Indianapolis, Indianapolis, IN, United States

**Keywords:** *Stenotrophomonas maltophilia*, antibiotic, biofilm, virulence, chronic infection, opportunistic

## Abstract

The Gram-negative pathogen *Stenotrophomonas maltophilia* causes a wide range of human infections. It causes particularly serious lung infections in individuals with cystic fibrosis, leading to high mortality rates. This pathogen is resistant to most known antibiotics and harbors a plethora of virulence factors, including lytic enzymes and serine proteases, that cause acute infection in host organisms. *S. maltophilia* also establishes chronic infections through biofilm formation. The biofilm environment protects the bacteria from external threats and harsh conditions and is therefore vital for the long-term pathogenesis of the microbe. While studies have identified several genes that mediate *S. maltophilia’s* initial colonization and biofilm formation, the cascade of events initiated by these factors is poorly understood. Consequently, understanding these and other virulence factors can yield exciting new targets for novel therapeutics.

## 1 Introduction

*Stenotrophomonas maltophilia* is an opportunistic pathogen emerging globally as a multidrug-resistant organism (Brooke et al., 2017). It causes severe nosocomial infections, such as pneumonia, bacteremia, endocarditis, meningitis, mastoiditis, biliary sepsis, and catheter-related urinary tract infections (Looney et al., 2009; Chang et al., 2015). This microbe causes particularly severe infections in immunocompromised individuals, intensive care unit (ICU) patients, those taking high doses of antibiotics, burn patients, cancer patients, and patients with transplants (Nseir et al., 2006a; Demiraslan et al., 2013; Hofmann et al., 2016; Adegoke et al., 2017). A 10-year case study from 2008 to 2017 showed a ~162% increase in the rate of *S. maltophilia* isolates from individuals with lower respiratory tract infection (Gajdacs and Urban, 2019). It is reported that there is a higher risk of transmission of infection through contaminated healthcare associated water supplies, such as tap water, hemodialysis water, and dental unit reservoirs (Cervia et al., 2008). Notably, S. *maltophilia* infections exhibit a mortality rate of up to 69% (Senol et al., 2002), with higher incidences in patients with chronic kidney diseases and foley catheter usage (Jian et al., 2022). *S. maltophilia* also causes severe lung infection and pulmonary exacerbation (Talmaciu et al., 2000; Waters et al., 2013; Berdah et al., 2018) in the lungs of 11.9-14% of individuals with cystic fibrosis (CF) (Ewig et al., 2000; Nseir et al., 2006b; Trifonova and Strateva, 2019a; McCutcheon and Dennis, 2021). Despite the wide range of infections it can cause, there is very little known about the immune response to *S. maltophilia* infection. Different *S. maltophilia* strains show different degrees of survival inside immature dendritic cells (iDCs) (Roscetto et al., 2015). They plausibly also evade phagocytosis of these effector cells, rendering these cells incapable of antigen presentation and immune activation along with utilizing the cells as a point of bacterial dissemination for infection spread.

## 2 *S. maltophilia* infection in individuals with cystic fibrosis

The most well studied *S. maltophilia* infection is in the lungs of CF individuals. CF is a genetic disorder resulting from point mutation of the cystic fibrosis transmembrane conductance regulator (CFTR) gene (Knowles and Durie, 2002). Over 160,000 people worldwide are known to be suffering from CF (Guo et al., 2022). These individuals experience defective clearance of the bronchial airways and bronchopulmonary system, causing build-up of mucus in the lungs (Gardner, 2007). They eventually experience abnormal respiratory inflammation, increased mucus deposition, and periodic bacterial infections. Thus, individuals with CF have symptoms like wheezing, coughing, and shortness of breath. More than 95% of these patients die due to pulmonary disease (Gardner, 2007).

*S. maltophilia* is commonly isolated from the airways of CF patients (Hansen, 2012). There has been a steady increase in incidence of *S. maltophilia* infection associated with CF individuals over the last decade (Amin and Waters, 2016). Reports indicate that mortality or lung transplantation is 3-fold higher in CF individuals with chronic *S. maltophilia* infection (Gallagher et al., 2019). It is possible that the high intrinsic antibiotic resistance of *S. maltophilia* provides it a colonization advantage in the CF lung, as these individuals are treated with large doses of many antibiotics. The weakened immune system of these individuals can also be a risk factor for *S. maltophilia* infection. In any case, *S. maltophilia* can be isolated at high levels (10^5^-10^6^ CFU/mL), suggesting deterioration of the individuals’ pulmonary function (Ballestero et al., 1995; Colin and Rabin, 2011). In fact, *S. maltophilia* infections are associated with lower forced expiratory volume (FEV1) (47.06%) than in individuals without this bacterium (73.40%), indicating rapid deterioration of lung function (Waters et al., 2011). Studies have also shown respiratory tract co-infections of *S. maltophilia* with *P. aeruginosa*, *Burkholderia cepacia*, methicillin resistant *Staphylococcus aureus* (MRSA), methicillin sensitive *S. aureus* (MSSA), *Aspergillus* spp, *Candida* spp, or *Achromobacter xylosoxidans* (Goss et al., 2002; Colin and Rabin, 2011; Granchelli et al., 2018).

Very little research has been done on the immune responses to *S. maltophilia* bacterial infection in CF persons. Chronic *S. maltophilia* infection in CF airways presents with measurable immunological reaction (production of anti- extracellular protease and antiflagellin antibodies) (Waters et al., 2011; Hansen, 2012). Higher antibody levels have also been found in the sera of CF individuals chronically infected with *S. maltophilia* than CF individuals with intermittent or no infection (Brooke et al., 2017). Apart from this, it is said that the immunological response of *S. maltophilia* towards the pulmonary infection in CF patients is quite similar to that of *P. aeruginosa* (Hansen, 2012). Experimentally, mice infected with *S. maltophilia* via a nebulizer exhibited higher IL-6, IL-12, IL1β, IFN-γ, and TNF-α cytokine levels and lower IL-4 levels early in infection, compared to mock infected mice (Di Bonaventura et al., 2010). Later in infection, only IFN-γ was found to be significantly higher than any other cytokines in the infected mice. There were also higher keratinocyte-derived cytokine (GROα/KC), monocyte chemotactic protein 1 (MCP-1/JE), macrophage chemoattractant protein 5 (MCP-5), macrophage inflammatory protein 1α (MIP-1α), macrophage inflammatory protein 2 (MIP-2), and thymus and activation regulated chemokine (TARC) levels during early infection in the infected mice than in the control (Di Bonaventura et al., 2010). Although the studies discussed here were not with a murine model of CF, they explore the dynamic immune response that could be observed for CF based on the infection response in these mice. It will be important for future studies to elucidate this response and comprehend the complex interactions of this bacterium in a CF lung environment.

## 3 Antibiotic resistance

One of the most confounding features of *S. maltophilia* is its strong resistance toward many antibiotics, including aminoglycosides, fluroquinolones, β-lactams, cephalosporins, macrolides, carbapenems, chloramphenicol, tetracyclines, polymyxins, and sulfonamides (Brooke, 2012). Of particular concern pan-resistant strains, which are resistant to nearly all known antibiotics, have been documented (Valdezate et al., 2001). In general, though, combinational therapy of trimethoprim sulfamethoxazole (TMP-SMX) is an effective treatment (Muder et al., 1996; Muder, 2007; Nicodemo and Paez, 2007; Goldberg and Bishara, 2012). A study in Taiwan, showed that 20.5% of patients with *S. maltophilia* infections exhibited TMP-SMX resistance (Wang et al., 2017). Additionally, all tested fluoroquinolones were seen to effectively reduce *S. maltophilia* infection at one half of the MIC (Di Bonaventura et al., 2004). Similarly, in one study of patients suffering from sepsis caused by *S. maltophilia*, levofloxacin worked well in 70.5% of the patients (Soumya et al., 2020). However, prior levofloxacin use may induce increased *S. maltophilia* resistance to TMP-SMX (Wang et al., 2017).

Recently, many antibiotic resistant *S. maltophilia* infections have been seen in patients with certain co-morbidities. For instance, ventilator associated pneumonia patients infected with *S. maltophilia* showed resistance to carboxypenicillin and carbapenem (Ibn Saied et al., 2020). A case study of a 70 year old woman with hypoglycemia, chronic respiratory failure, adenocarcinoma of the lung, anemia, and other co-morbidities reported the presence of *S. maltophilia* in the respiratory tract; this infection showed resistance to broad spectrum antibiotics except for TMP-SMX and levofloxacin (Kanderi et al., 2020). These cases, and many others, indicate that new treatments are needed to treat this uncompromising, deadly pathogen.

### 3.1 Mechanism of antibiotic resistance

*S. maltophilia* displays antibiotic resistance through several mechanisms. *S. maltophilia* clinical isolate K279a carries nine Resistance Nodulation Division (RND)-type efflux pump genes that confer resistance to a number of different antibiotics (Crossman et al., 2008). For example, efflux pump operon *smeABC* contributes greatly to antimicrobial resistance, due to production of the outer membrane efflux lipoprotein *SmeC (* Li et al., 2002 *)*. Also, over-expression of *smeDEF* induces tetracycline, erythromycin, and fluoroquinolone resistance (Alonso and Martinez, 2000). Among the rest of the other RND efflux pumps genes, *smeZ*, *smeJ*, and *smeK* each contribute to aminoglycoside, fluoroquinolone, and tetracycline resistance (Gould et al., 2013). It was also seen that *smeYZ* conferred aminoglycoside and TMP-SMX resistance (Lin et al., 2015). Apart from this, a fusaric acid extrusion efflux pump (FuaABC), an MSF type efflux pump (EmrCABsm), and two ABC-type efflux pumps (SmrA, MacABCsm) confer antimicrobial resistance (Al-Hamad et al., 2009; Hu et al., 2012; Huang et al., 2013). Some strains of *S. maltophilia* encode *floR*, a gene found in insertion element common region (ISCR) adjacent sequences that aids in phenicol class of antibiotic resistance via expression of an exporter (Toleman et al., 2007).

In addition to efflux pumps, several antibiotic modifying enzymes make this bacterium resistant to many antimicrobials. L1 and L2 β-lactamases produced by *S. maltophilia* hydrolyse β-lactams (Avison et al., 2001). The L1 enzyme is a metallo-β-lactamase conferring resistance to all β-lactams, including penicillins, cephalosporins, and carbapenems (Saino et al., 1982; Paton et al., 1994; Walsh et al., 1994; Crowder et al., 1998), whereas L2 is a cephalosporinase (Saino et al., 1984; Walsh et al., 1997). *S. maltophilia* also carries an *mphBM* gene, which displays high homology to a macrolide phosphotransferase in *S. aureus* that makes *S. aureus* resistant to erythromycin (Alonso et al., 2000). It is possible that this factor does the same in *S. maltophilia*. Presence of *sul1*, *sul2*, and the ISCR element have been found in some strains of *S. maltophilia* rendering them resistant to TMP-SMX and other sulfonamides (Barbolla et al., 2004; Toleman et al., 2007; Chung et al., 2015). *dfr* genes expressing the enzymes dihydrofolate reductase has been also helpful in making strains resistant to trimethoprim. Thus both *sul* and *dfr* genes are seen in the TMP-SMX resistance strains (Hu et al., 2011; Hu et al., 2016). Other resistance genes have also been found in ISCR adjacent sequences such as *dhf*rA10, *dhf*rA9, *dhf*rA20 (aiding in trimethoprim resistance), *tetR* (aiding in resistance to the tetracycline class), and *strA* (aiding in streptomycin resistance) (Toleman et al., 2007). Interestingly, research has shown that some innately resistant environmental bacteria, such as *S. maltophilia*, can metabolize the carbon from antimicrobial agents to aid in growth (Martinez, 2008) (Dantas et al., 2008). Additionally, a truncated phosphoglucosamine mutase, encoded by *glmM*, indirectly counters the effects of antibiotics that target bacterial cell wall peptidoglycan (Avison et al., 2001), and the *qnr* gene protects bacteria from quinolone antibiotics targeting DNA gyrase and topoisomerase (Zhang et al., 2023). However, prevalence of most of these resistance factors is unknown.

## 4 Metal resistance

*S. maltophilia* can also grow in the presence of most heavy metals such as copper, zinc, cobalt, nickel, mercury, silver, antimony, tellurite, selenite, lead, and molybdenum among other metals (Pages et al., 2008; Deredjian et al., 2016; Yu et al., 2018; Zhang et al., 2023). For instance, *S. maltophilia* strain D457R, a multiresistant derivative of clinical isolate D457, displays cadmium resistance due to the cadmium efflux pump encoded by *cadA* (Pages et al., 2008). *cadC*, a regulator of *cadA* gene expression aids in making the bacteria cadmium resistant (Alonso et al., 2000; Pages et al., 2008). *S. maltophilia* was seen to express a SulP family protein, potentially involved in sulfate permease activities responsible for uptake of oxyanions such as selenate and selenite (Yu et al., 2018). A phosphate uptake protein with a PitA-like sequence had also been found in *S. maltophilia* conferring arsenate resistance. Genes putatively encoding homologs of metal transporters MgtA, MgtE, CorA, MntH, and ZupT have been identified, and these potential transporters can cause accumulation of Mg²^+^, Mn²^+^, Ni²^+^, Zn²^+^, Cd²^+^, Co²^+^, and Cu²^+^ (Yu et al., 2018). Additionally, genome annotations have revealed multicopper oxidases and copper transporting, binding, and storage proteins conferring bacterial resistance to copper. Furthermore, genome analysis has revealed MdtABC efflux pumps of zinc; CzcA, CzcB, and CzcC proteins causing efflux of zinc, cadmium, and cobalt; and ArsC, arsenite reductase, mercury reductase, and other arsenite and mercury transporter proteins conferring arsenic and mercury resistance (Yu et al., 2018). This metal resistance, combined with the extreme antibiotic resistance that *S. maltophilia* displays, makes this pathogen extremely difficult to kill (Edet et al., 2023).

## 5 Virulence factors

Though traditionally considered a low virulence pathogen, *S. maltophilia* contains genes with homology to numerous classical virulence factors, though many have not been experimentally validated (Trifonova and Strateva, 2019a). Here, we highlight the vast arsenal that *S. maltophilia* contains with which it conducts pathogenesis.

### 5.1 Lytic enzymes

**Figure 1:**
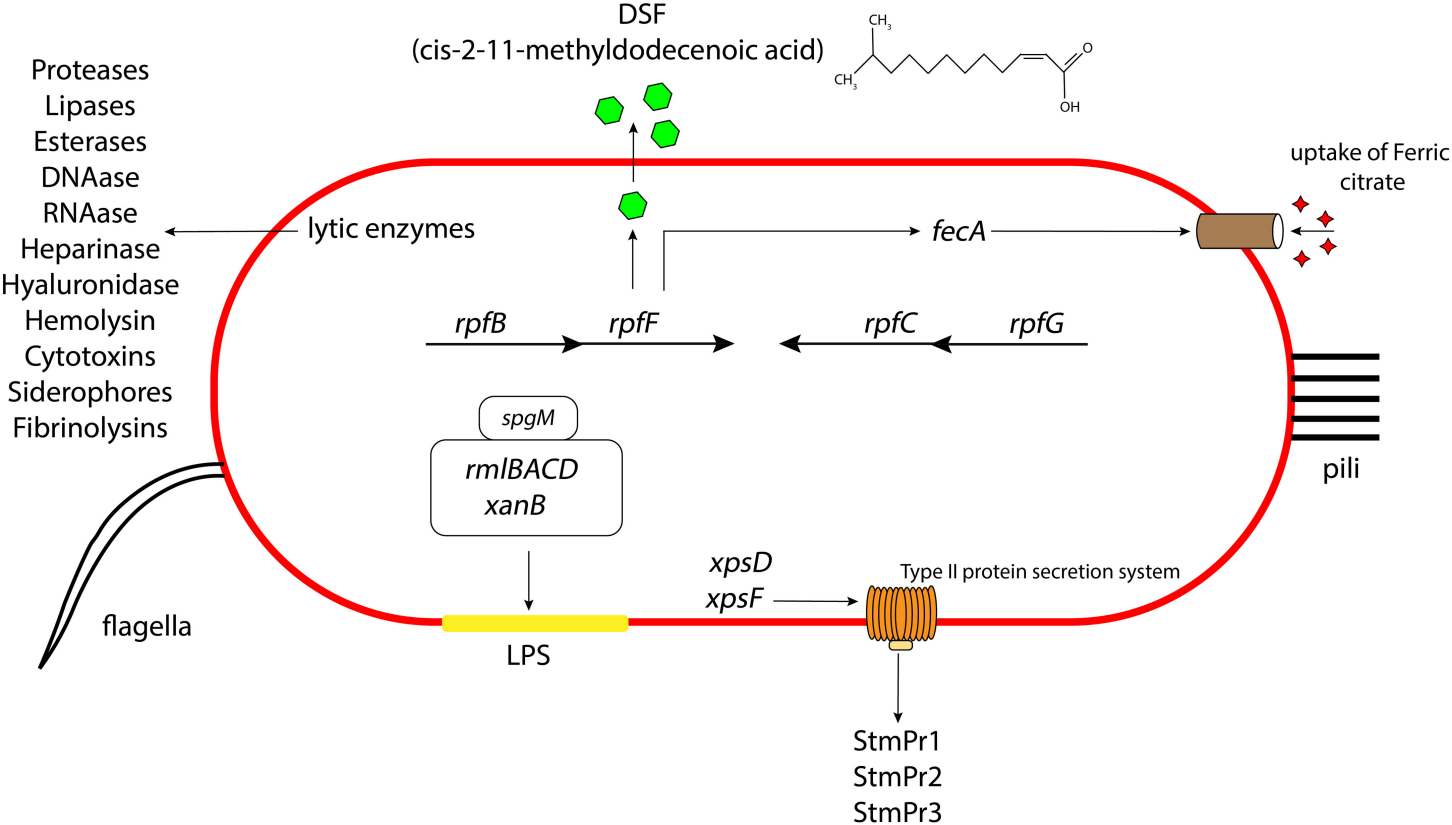
Schematic representation of the different virulence and attachment factors in *S. maltophilia*. The role of LPS, lytic enzymes, quorum sensing, secretion systems, iron uptake mechanisms contributing to the overall bacterial pathogenesis, along with the presence of pili and flagella aiding in their initial attachment and colonization. Not drawn to scale.

*S. maltophilia* can secrete a variety of different extracellular enzymes, including phospholipase, DNAase, RNAse, esterases, gelatinase, lipases, proteinase, proteases, heparinase, hyaluronidase, and hemolysin (Crossman et al., 2008; Thomas et al., 2014) (**Figure 1**). Serine proteases StmPr1, StmPr2, and StmPr3, have particularly been shown to make *S. maltophilia* highly cytotoxic. Among other effects, these proteases sever the host cell extracellular matrix (ECM) proteins like human type I collagen, fibrinogen, and fibronectin (DuMont and Cianciotto, 2017). These proteases also show caseinolytic, gelatinase, and hydrolytic activities, and they are associated with loss of mammalian cell structural viability, changes in actin cytoskeleton, loss of integrin/ECM connections, cell detachment, and degradation of IL-8. These effects can trigger the process of apoptosis (anoikis) by activating a cascade of caspases starting from caspase 3, 6, and 7 (DuMont and Cianciotto, 2017).

### 5.2 Secretion systems

*S. maltophilia* also produces type I, type II, type IV, type V, type VI secretion systems. Many of the above lytic enzymes are secreted through one of these apparatuses. For instance, the StmPr proteases, secreted through the *xps*-encoded type II secretion (T2S) system, causes cell rounding, actin rearrangement, degradation of extracellular matrix proteins, degradation of IL-8, cell detachment, and ultimately cell death of alveolar epithelial cells (Karaba et al., 2013; DuMont et al., 2015; DuMont and Cianciotto, 2017) (**Figure 1**). *S. maltophilia* also express a type IV secretion system (T4SS) whose effector molecules render the pathogen advantage in interbacterial competition with *P. aeruginosa* (Nas et al., 2021) and also lyses infected mammalian macrophages (Nas et al., 2019). It is suggested that the VirB/D4 apparatus of T4SS has pro- and anti-killing effector molecules which have disparate actions on normal and infected mammalian cells and heterologous bacteria inhabiting the human host (Nas et al., 2019). Another study suggests that there are two effector molecules of the T4SS in *S. maltophilia*, namely TcfA and TcfB, which on expression kill the target bacterial cell in a mixed bacterial environment (Nas et al., 2021). Based on assays, sequence analysis of the effector protein and structural prediction, it is hypothesized that in a coculture environment, TcfA, a lipase enzyme, on injection to the outer or inner membrane of the targeted competitor cell causes cell death. Sequence similarity and software prediction also shows that TcfB is anticipated to belong to a family of endolysins that exhibit lethal properties linked to an activity resembling lysozyme. It is hypothesized to interact and degrade the peptidoglycan for effective killing of the targeted cell. *S. maltophilia* environmental and clinical isolates also possess a type VI secretion system (T6SS) (Crisan et al., 2023). Genome annotation suggest that this system expresses proteins with putative phospholipase, endopeptidase, and lysozyme like activity. There is also evidence that pulmonary *S. maltophilia* strain STEN00241 can utilize a T6SS to compete with *P. aeruginosa*, and outcompete *B. cenocepacia* and *E. coli* in a co-culture environment (Crisan et al., 2023). Finally, genome annotations indicate the presence of type I secretion systems in strains K279a and R551-3 (Rocco et al., 2009) and type V secretion system (autotransporter) in strain K279a (Crossman et al., 2008). Intriguingly, this Gram-negative pathogen does not make a type III secretion system.

### 5.3 Diffusible signaling factor

Pathogenesis in *S. maltophilia* is governed by a quorum sensing molecule called diffusible signaling factor (DSF), or *cis*-11-methyl-2-dodecenoic acid. DSF synthesis is encoded by *rpfF* (An and Tang, 2018) (**Figure 1**). *rpfF* is transcribed as part of an operon with *rpfB*, encoding a long chain fatty acyl coA ligase (on which the secretion of DSF is partially dependent) (Yero et al., 2020). A convergently transcribed operon consisting of *rpfC* and *rpfG* encodes a 2-component system of sensor kinase and response regulator, respectively, which are involved in DSF transcriptional regulation. DSF has been shown to regulate *S. maltophilia* biofilm formation, synthesis of extracellular polymeric substance (EPS), and secretion of protease (Huang et al., 2006; Ryan et al., 2015; Yero et al., 2020). It also contributes to bacterial motility. Furthermore, DSF was seen to influence the production of β-lactamase, making the bacterium resistant to β-lactams. This finding was the first evidence for the role of DSF signaling in antibiotic resistance (Alcaraz et al., 2019). DSF can also regulate the expression of an outer membrane receptor FecA, used for the uptake of ferric citrate in *S. maltophilia (* Matera et al., 2004). Some *S. maltophilia* strains contain an extra DSF-related gene, *rpfS*, which contributes to epiphytic disease (An et al., 2014). Intriguingly, DSF also mediates interspecies communication and can thus mediate coculture of *S. maltophilia* with other species (Ryan et al., 2015).

### 5.4 Lipopolysaccharide

The LPS layer of Gram-negative bacteria, including *S. maltophilia*, can greatly influence the antimicrobial activities of the cell. The LPS in *S. maltophilia* is primarily made of O-specific polymer formed of D-glucose, L-rhamnose, and D-fucose (Winn et al., 1993). The endotoxicity of the LPS layer is associated with neutrophil activation or IL-8 production inside the host which gives rise to a systemic immune response. Various combinations of antibiotics interacting with the LPS could increase (ceftazidime) or decrease (β-lactams and aminoglycosides) this immune response (Matera et al., 2004). In *S. maltophilia*, the *spgM* gene, encoding for phosphoglucomutase and phosphomannomutase enzyme activities (like the phosphoglucomutase producing gene *algC* in *P. aeruginosa*), contributes to the production of a thicker LPS layer (McKay et al., 2003) (**Figure 1**). A strain with mutation of the *spgM* gene exhibited a two to four fold increased susceptibility to aminoglycosides, quinolones, vancomycin, polymyxin B and E, and many other antibiotics. The *spgM* mutant was also unable to colonize rat lungs, suggesting that the gene is an important factor in the virulence of the bacterium. Other genes affecting *S. maltophilia* LPS production include *xanB*, *rmlA*, and *rmlC* (Huang et al., 2006). Mutation of *rmlAC* and *xanB* results in lowered O-antigen production in the bacterial LPS, leading to a defective outer membrane layer and impaired flagellar production, motility, adherence, and biofilm formation (Huang et al., 2006).

### 5.5 Attachment factors

#### 5.5.1 Pili

Microscopy studies have demonstrated the presence of peritrichous semiflexible pili on the surface of *S. maltophilia* (de Oliveira-Garcia et al., 2003) (**Figure 1**). These pili have been named *S. maltophilia* fimbriae-1, or Smf-1. Electron microscopy images reveal that Smf-1 has a size of 5-7nm in width and forms primarily at 37°C (Flores-Trevino et al., 2019; Kalidasan and Neela, 2020). The Smf-1 N-terminal amino acid sequence is similar to that of the CupA fimbriae in *P*. *aeruginosa* and similar chaperone/usher pili in other Gram-negative bacteria (de Oliveira-Garcia et al., 2003). Smf-1 is capable of agglutinating red blood cells from several animals, including humans (de Oliveira-Garcia et al., 2003). It also binds to several abiotic and biotic surfaces, such as medical devices and human epithelial cells (de Oliveira-Garcia et al., 2003). Additionally, this pilus is highly immunogenic. Mice administered purified Smf-1 showed a high innate immune response with increase in pro-inflammatory cytokines (*e.g.* IL-1β, and TNF-α). These mice also exhibited elevated IL-8 levels in the bladder, suggesting neutrophil chemotaxis, followed by high nitric oxide production (Zgair and Al-Adressi, 2013).

Through genome annotations, it has been reported that *S. maltophilia* expresses a type I pilus that is assembled via the chaperone usher pathway (Rocco et al., 2009). These types of pili are comprised of a shaft made of a primary fimbrial protein and two other ancillary proteins. This *S. maltophilia* pilus has been linked to bacterial adhesion and initial stages of biofilm formation (Ryan et al., 2009).

Type IV pili also play a role in *S. maltophilia* virulence (Trifonova and Strateva, 2019b). Type IV pili in *S. maltophilia* CF isolates contribute to the bacterial biofilm development (Pompilio et al., 2011). They also putatively mediate adherence to surfaces, auto-aggregation, and twitching motility of the bacterium (Ryan et al., 2009). Importantly, the *S. maltophilia* type IV pilus is also a receptor for several bacteriophages such as DLP1 and DLP2 (McCutcheon et al., 2018). Additionally, AXL3 bacteriophage has been shown to interact with the PilA subunit present in the type IV pilus rod, and the subsequent viral penetration is facilitated as the physical retraction of the type IV pilus allows the attached phage to reach the cell surface (McCutcheon et al., 2020). If we can understand and control this interaction, it is possible that this mechanism can be exploited in developing specialized tools and treatments tailored to fight this multidrug resistant pathogen.

#### 5.5.2 Flagella

*S. maltophilia* produces a polar flagellum, which is considered a significant attachment factor aiding colonization of host cells (**Figure 1**). The main structural protein of *S. maltophilia* flagella is a 38kDa protein called FliC (de Oliveira-Garcia et al., 2002). Studies have shown that, in several clinical isolates of *S. maltophilia*, presence of flagella correlates with binding to mouse tracheal mucus (Zgair and Chhibber, 2011). The flagellin protein acts as an adhesin, and anti-flagellin antibody significantly lowers bacterial adherence to mucus. Additionally, a homolog of the *Xanthomonas campestris flil* gene (encoding a flagellin associated ATPase) demonstrated an important role in flagellar production and bacterial colonization of *S. maltophilia* strains isolated from individuals with cystic fibrosis (Di Bonaventura et al., 2007). Another study showed that mutation of the *ompA* gene (encoding a porin protein) severely compromised the integrity of the *S. maltophilia* outer membrane. This action caused a defect in flagellar assembly which resulted in failure of bacterial swimming motility (Liao et al., 2021). In *S. maltophilia*, BsmR (biofilm and swimming motility regulator) regulates the expression of 349 genes, including *fsnR*, which in turn switches on the expression of 2 operons required for flagella production (Kang et al., 2015; Liu et al., 2017). With the activation of these operons, flagella production is increased, which increases the bacterial motility.

#### 5.5.3 Phosphoglycerate mutase impacts bacterial attachment

A recent genetic screen of *S. maltophilia* strain K279a identified *gpmA*, encoding glycolytic enzyme phosphoglycerate mutase, as affecting biofilm formation (Ramos-Hegazy et al., 2020). Subsequent isogenic deletion of *gpmA* resulted in defects in adhesion to biotic and abiotic surfaces. Initial attachment of the Δ*gpmA* strain was greatly reduced at early time points compared to the wild type strain on polystyrene plates. Nevertheless, there was no difference in biofilm formation at later time points, which suggests that this gene is required for early attachment and development of biofilms on abiotic surfaces. It was also observed that the Δ*gpmA* strain exhibited a 100-fold reduction in binding to CF bronchial epithelial cells (CFBE) compared to the wild type strain at 1 hour. As with polystyrene plate biofilms, though, there was no significant difference between the wild type and Δ*gpmA* strains at later time points. It is unclear why a metabolic gene affects attachment, though this finding suggests an intriguing link between metabolism and virulence in *S. maltophilia.*

### 5.6 Other virulence factors

Genome annotation show that *S. maltophilia* strain K279a also produces a YadA-like protein, of the BuHA family of proteins (Nseir et al., 2006a). First identified in *Burkholderia mallei* (Tiyawisutsri et al., 2007), members of this protein family have been shown to have filamentous hemagglutinin, invasin, and hemolysin properties, and they are considered as important virulence factors in the pathogenesis and rapid spread of bacteria (Colombi et al., 2006; Holden et al., 2009). Indeed reports mention the presence of filamentous hemagglutinins via genome annotation in various environmental and clinical strains of *S. maltophilia* that conduct virulence through cell to cell aggregation (Ryan et al., 2009). *S. maltophilia* strains were also found to produce outer membrane vesicles that are cytotoxic to alveolar epithelial cells and induce a strong inflammatory response in both *in vitro* and *in vivo* environments (Kim et al., 2016). Additionally, the bacterium can protect itself from host defenses through expression of enzymes such as alkyl hydroperoxidase, superoxide dismutase, catalase, and melanin pigments that can disrupt or counteract host defense products (Thomas et al., 2014; Jair et al., 2019; Li et al., 2020). Southern blot hybridization and PCR analysis have shown *S. maltophilia* to carry a phage gene sequence mildly homologous to a sequence in *Vibrio cholerae* producing the zonula occludens toxin (zot) (Hagemann et al., 2006). This toxin impairs the intercellular junctions in host cells allowing easy access for infection (Fasano et al., 1991).

## 6 *S. maltophilia* biofilm formation

Most microorganisms in nature attach to and grow on surfaces (Armbruster and Parsek, 2018). This attachment facilitates the aggregation of the bacteria into microcolonies, which increase in size as bacteria multiply and other cells attach to the cluster. The constituent microbial cells produce a polymer matrix that surrounds the cluster, creating a slimy layer. This process is called biofilm formation (Hernández-Jiménez et al., 2013; Deepigaa, 2017). Bacteria generally remain in sessile communities within the biofilm, as the biofilm matures (Crouzet et al., 2014). Eventually, as the biofilm ages, cells disperse and spread to other locations. Because of the chemical and physical conditions within biofilms, the bacterial community is protected from adverse environmental conditions, such as extreme temperatures and extreme pH, high salt concentrations, UV radiation, depletion of nutrients, host immune responses, antimicrobial agents, peptides (LL37, β-defensins, dermcidin, for example), and even antibiotics (Hoiby et al., 2010; Yasir et al., 2018; Yin et al., 2019). Biofilms can be found in nature, in medical settings, and in industrial settings (Mah and O’Toole, 2001; Hall-Stoodley et al., 2012), and during infection, biofilms contribute to pathogenesis due to their highly resistant nature (Yin et al., 2019). Some estimates indicate that in humans, about 60-80% of microbial infections involve a biofilm component (Costerton et al., 1999). Thus, it is important to understand the pathways that trigger this physiological process so we can better fight the causative organisms.

*S. maltophilia* is a known biofilm producer. In one study, 98.7% of 150 clinical *S. maltophilia* isolates produced biofilm (Azimi et al., 2020). Among this percentage, 46% had strong biofilm producing capabilities. In a few molecular studies of *S. maltophilia* biofilm, *smf-1*, *rmlA*, *rpfF*, and *spgM* genes were found to influence *S. maltophilia* biofilm initiation and development (Azimi et al., 2020); these genes are widespread among *S. maltophilia* strains (Azimi et al., 2020).

Additionally, studies have shown that iron regulation in *S. maltophilia* influences biofilm formation (García et al., 2015). For instance, the Fur protein regulator that maintains iron homeostasis in the cell also regulates certain virulence factors and mediates oxidative stress response (Kalidasan et al., 2018a). Isogenic deletion of the *fur* gene results in decreased biofilm in iron depleted conditions, compared to iron-rich environments. Moreover, upregulation of two iron repressed outer membrane proteins (IROMPs) in such conditions also affects biofilm. These proteins are putatively required for scavenging iron from the environment to induce higher biofilm growth.

While *S. maltophilia* strains producing strong biofilms on abiotic surfaces show significant resistance to many antibiotics, like ticarcillin clavulanate, ceftazidime, ciprofloxacin, and doxycycline (Azimi et al., 2020), some antibiotics have been found to be quite effective in treating biofilms of this microbe. For instance, TMP-SMX was found to be active in reducing bacterial viability of *S. maltophilia* in biofilm (Di Bonaventura et al., 2004). The fluoroquinolone moxifloxacin was also useful in stopping bacterial adherence, by inducing cell lysis, resulting in detachment of bacterial cell surface glycocalyx from polystyrene surfaces (Di Bonaventura et al., 2004). Another study showed that levofloxacin could inhibit biofilm formation of clinical *S. maltophilia* strains in the presence of erythromycin (Sun et al., 2016). Erythromycin can also act synergistically with cefoperazone/sulbactam, and piperacillin in increasing susceptibility among constituents of biofilms (Sun et al., 2016). This indicates a potential use of combination therapy of certain antibiotics with macrolides in combating *S. maltophilia* biofilm formation.

Interestingly, one study found an inverse relationship between antibiotic resistance and the biofilm formation of *S. maltophilia* (Pompilio et al., 2020). It was seen that pathogenic strains of *S. maltophilia* susceptible to ceftazidime, levofloxacin, colistin, and ticarcillin clavulanate showed greater biofilm formation capabilities than pathogenic strains resistant to the same antibiotic. However, there was no significant difference in the level of biofilm for non-pathogenic *S. maltophilia* strains for any antibiotic (Pompilio et al., 2020). Studies also showed mutation of *smeYZ* genes, conferring aminoglycoside and TMP-SMX resistance to the bacteria, produced lesser biofilm than the wild type clinical isolate *S. maltophilia* KJ (Lin et al., 2015).

## 7 *S. maltophilia* and metabolism

**Figure 2:**
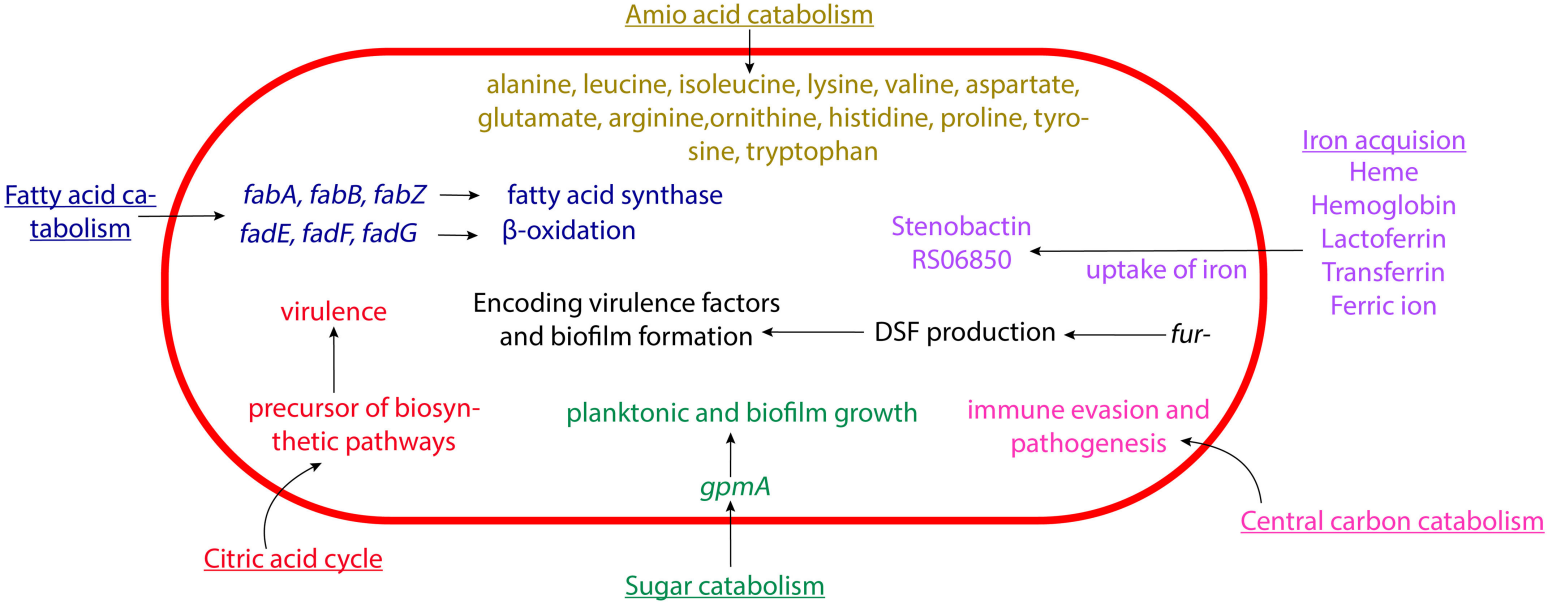
Schematic representation of the metabolism of *S. maltophilia* affecting its pathogenesis. The fatty acid catabolism, citric acid cycle, sugar catabolism, amino acid catabolism, central carbon catabolism, and iron acquisition come together in contributing to the overall bacterial virulence. Not drawn to scale.

The name “Stenotrophomonas” means “narrow eating unit”, which refers to the comparative lack of substrates utilized by this microorganism for growth. “Maltophilia” means “maltose lover” (Denton and Kerr, 1998). Historically, it has been thought that this pathogen mainly utilizes hexose sugars, and growth studies somewhat support that assumption. Beyond sugar, though, *S. maltophilia* displays some intriguing metabolic characteristics. In fact, recent studies have made connections between *S. maltophilia* metabolism and virulence behaviors, including biofilm formation (**Figure 2**).

Iron acquisition has an important role in *S. maltophilia* metabolism (Kalidasan et al., 2018b). This includes the use of siderophores like enterobactin and stenobactin, alongside multiple other pathways that can utilize various iron sources (Ryan et al., 2009; Adamek et al., 2014; Suvorova and Rodionov, 2016; Nas and Cianciotto, 2017; Kalidasan et al., 2018b; Azman et al., 2019; Brooke, 2021). The microbes’s central carbon catabolism is crucial for biosynthesis and energy production (Munoz-Elias and McKinney, 2006). *S. maltophilia* contains genes for several glycolytic pathway, including Embden-Meyerhof-Parnas, Entner-Doudoroff, and pentose phosphate pathways (Stanier et al., 1966; Munoz-Elias and McKinney, 2006; Crossman et al., 2008). As mentioned above, glycolytic gene *gpmA* appears to affect *S. maltophilia* attachment. *gpmA* encodes phosphoglycerate mutase, which catalyzes the interconversion of 2-phospho-D-glycerate and 3-phospho-D-glycerate. It was further discovered that without *gpmA, S. maltophilia* planktonic growth and biofilm formation are severely decreased when grown on different substrates (Isom et al., 2022; Ramos-Hegazy et al., 2020). Intriguingly, recent work suggested that under low nutrient conditions this pathogen preferentially utilizes amino acid over glucose as carbon source, potentially shuttling carbons through GpmA (Isom et al., 2022). Beyond iron acquisition pathways and glycolysis, *S. maltophilia* contains genes for the citric acid cycle (Kanehisa et al., 2023), β-oxidation and fatty acid synthesis (Haydon and Guest, 1991; Heath and Rock, 1996; Rock and Cronan, 1996; Textor et al., 1997; Cronan and Subrahmanyam, 1998; DiRusso and Nyström, 1998; Wang and Cronan, 2004; Fujita et al., 2007; Shuman, 2010; Kanehisa et al., 2023), and utilization of various amino acids like alanine, leucine, isoleucine, lysine, valine, aspartate, glutamate, arginine, ornithine, histidine, proline, tyrosine, and tryptophan (Omenn, 2010). Investigating the possible connection of these metabolic pathways to pathogenesis will be an intriguing and potentially fruitful avenue of research in the future.

## 8 Conclusion

*S. maltophilia* is a fascinating microorganism that presents us with extensive challenges and opportunities. Because of the lack of knowledge of its virulence mechanisms and basic biology, there is a huge potential for further research to understand the evolution, adaptability, and the versatility of this pathogen. It is evident that we know quite a bit about the antibiotic resistance of *S. maltophilia*, the types of virulence factors it can produce, and its role in infection of the bronchopulmonary system. Additionally, much work has been done regarding *S. maltophilia* multi-species biofilm, roles of iron, DSF factor, different lytic enzymes, and treatment options for CF individuals colonized with this microbe, but very little work has been done to understand the mechanism of biofilm formation, the regulators in biofilm growth, the specific immune responses to the formation of *S. maltophilia* biofilm, and the cascade of virulence factor events during an infection. An integrated disease model should be established to develop newer understanding into such various topics. These efforts may aid in understanding infection patterns and pathogenesis and yield insights about novel molecular and cellular drug targets. Moreover, greater efforts should also be placed on surveillance of the worldwide drug resistance situation and the role opportunistic pathogens like *S. maltophilia* play in genetic transfer of resistance determinants. Apart from this, climate change has been identified as a significant factor in altering the transmission patterns of *S. maltophilia*, rendering it an extremely hazardous pathogen (Lafferty, 2009; Omenn, 2010; Shuman, 2010; Brooke, 2012). According to current research, the global average temperature will increase 1.8°C to 5.8°C over the next 100 years and that may alter the hydrolytic cycle of the environment, increase pollution, and create poorer sanitation (Shuman, 2010). It is also expected that this will change the geographical distribution of waterborne *S. maltophilia*, alter its microbial evolution pattern, and give the bacterium a susceptible environment to increase its spread (Brooke, 2012; Brooke, 2021). Further investigation could ultimately lead us to update our knowledge about this emerging global opportunistic pathogen, *S. maltophilia*, and to identify novel therapies to inhibit its growth and spread.

## Author contributions

RB: Conceptualization, Writing – original draft, Writing – review & editing. NA: Conceptualization, Writing – original draft, Writing – review & editing. GA: Conceptualization, Writing – review & editing.
